# Durable Epoxy@ZnO Coating for Improvement of Hydrophobicity and Color Stability of Wood

**DOI:** 10.3390/polym11091388

**Published:** 2019-08-23

**Authors:** Vu Manh Tuong, Nguyen Van Huyen, Nguyen Trong Kien, Nguyen Van Dien

**Affiliations:** College of Wood Industry and Interior Design, Vietnam National University of Forestry, Ha Noi 156200, Vietnam

**Keywords:** coating, epoxy resin, micro/nanomaterials, superhydrophobic, *Styrax tonkinensis* wood, UV-resistant, water-resistant

## Abstract

The hydrophobicity and color stability of wood are important properties that can be easily changed when wood is used as a raw material for outdoor products, reducing the service life of wood. Herein, an epoxy@ZnO coating was applied by a two-step simple spray coating method to improve the hydrophobicity and color stability of *Styrax tonkinensis* wood. The hydrophobicity, robustness of coating, as well as the color stability of uncoated wood samples and epoxy@ZnO coated wood samples were evaluated. The microstructure morphology and crystal structures of the coating were also characterized by a field-emission scanning electron microscope (FESEM) and X-ray diffraction (XRD) analysis, respectively. Results showed that the obtained epoxy@ZnO coating was not only superhydrophobic with an average water contact angle of 154.1°, but also maintained superhydrophobicity with an average water contact angle of 149.6° after five water jetting tests. The color stability of the coated wood samples was improved by around 50% compared to that of uncoated wood samples. Additionally, a continuous epoxy@ZnO coating with hierarchical micro/nanoscale structures constructed by the wurtzite hexagonal structure of ZnO micro/nanoparticles on wood surfaces was confirmed.

## 1. Introduction

Wood is a natural, environmentally-friendly material used in many applications, such as furniture, construction, interior decoration, and outdoor products. However, when wood is used in outdoor applications, it is difficult to avoid exposure to water or moisture and ultraviolet (UV) rays from solar radiation. Consequently, wood surfaces weather easily, which may result in color change, cracking, dirt uptake, and damage of the wood microstructure during unprotected exposure. Moreover, the dimensional stability and exterior performance of wood, as well as its mechanical and physical properties, may be reduced because of contact with water [1,2]. Thus, construction of a coating with water-resistant and UV-resistant properties is an excellent solution to prevent or reduce the weathering problem. Such a coating will be useful in minimizing direct contact between wood and water, as well as ultraviolet rays from sunlight.

Recently, many studies have shown that coatings with hierarchical structures derived from surface roughness on the micrometer and nanometer scales, with low surface energy, can achieve superhydrophobicity and ultraviolet resistance [3,4,5,6,7,8]. In the case of wood nano-coating, there are several methods available, such as the sol-gel technique [9], the hydrothermal method [10,11], spray coating [12], layer-by-layer coating [13], solution-immersion [14], and cold plasma spraying [15].

Even though a great deal of research has been done to improve the water resistance and UV resistance of wood [16,17,18,19,20,21], it is still challenging to develop a novel facile method with cost-effective and long-lasting durability to meet practical requirements. Additionally, methods have been developed to fabricate superhydrophobic coatings on wood, mainly using inorganic compounds, such as TiO_2_, SiO_2,_ and ZnO, on the microscale or nanoscale. Zinc oxide (ZnO) with its numerous outstanding properties has been used broadly in various applications because of its low cost, non-toxicity, and high quantum yields [22]. Therefore, it is also an efficient material for outdoor wood surface coatings [23].

In order to find a facile and convenient coating method for large-scale production that can improve the hydrophobicity and color stability of wood, a two-step spray coating method was used in this research. Firstly, hydrophobic ZnO particles in micro/nano sizes were prepared by stearic acid modification of laboratory self-fabricated ZnO powder [24,25]. Secondly, as-prepared hydrophobic ZnO particles were applied on incompletely cured epoxy pre-coated wood using a spray gun. The hydrophobic ZnO particles play the role of forming a multiscale roughness, and incompletely cured epoxy resin pre-coating made the durability of the coating [26]. The microstructure of the epoxy@ZnO coating on wood surfaces and the crystal structure of the ZnO in the coating were evaluated by a field-emission scanning electron microscope (FESEM) and X-ray diffraction (XRD), respectively.

## 2. Materials and Methods

### 2.1. Materials

Main chemicals including zinc nitrate hexahydrate (Zn(NO_3_)_2_·6H_2_O, reagent grade, 98%), hexamethylenetetramine (C_6_H_12_N_4_, HMTA, reagent grade, 98%), stearic acid (CH_3_(CH_2_)_16_COOH, STA, reagent grade, >98%), epoxy resin (#3021, Part A and Part B), and solvent were supplied by Tianjin Baishi Chemical Industry Co., Ltd. (Tianjin, China) and were used as received without further purification. Wood samples were obtained from three plantation *Styrax tonkinensis* trees, each of which was six years old, that were collected from Tuyen Quang Province, Vietnam. Approximately the same amount of defect-free wood was cut around the same growth ring to prepare wood samples of 5 mm (T) × 30 mm (R) × 50 mm (L) in size (where R represents the radial direction, T represents the tangential direction, and L represents the longitudinal direction). Samples were separated into three groups (uncoated wood samples S1, ZnO-coated wood samples S2, and epoxy resin@ZnO-coated wood samples S3), and each treatment group contained a total of 10 test samples. All samples were placed in a chamber with a temperature of 20 °C and a relative humidity of 65% for three weeks before the superhydrophobic coating.

### 2.2. ZnO Micro/Nanoparticles Preparation and Hydrophobic Modification

The typical procedure used to prepare the ZnO micro/nanoparticles was based on the method of Wirunmongkol, et al. [27] with some modifications. Firstly, the equimolar aqueous solution (0.05 M) of zinc nitrate hexahydrate (Zn(NO_3_)_2_·6H_2_O) and hexamethylenetetramine (C_6_H_12_N_4_, HMTA) was prepared. Next, the as-prepared solution was poured into a Teflon-lined stainless-steel autoclave, which was then heat-treated at 90 °C for 4 h. The white precipitate was found in the solution. After that, the precipitate was separated from the solution by centrifugation at 2000 rpm for 15 min. The precipitate was washed with de-ionized water several times until the pH of the washed water became 7. The precipitate was oven-dried at 103 °C for twenty hours.

In order to produce the hydrophobic ZnO particles, the as-prepared ZnO particles were modified by STA. Typically, the ZnO micro/nanoparticles and STA were mixed in acetone with a weight ratio of 10:1 followed by constant stirring for 30 min at ambient temperature, and then dispersed in an ultrasonic washer for 10 min to obtain a white suspension [25]. The suspension was then used to spray coating on the wood substrate.

### 2.3. Superhydrophobic Coating Preparation

Epoxy #3021 solution of 50 wt% was prepared by mixing the two components (Part A and Part B) at a weight ratio of 1 to 1 in acetone. Then the as-prepared wood samples were immersed in the epoxy solution for 5 min. After that, the samples were dried at room temperature for 20 min. This process was repeated three times to obtain a continuous incompletely cured epoxy coating. Subsequently, the hydrophobically modified ZnO suspension was applied to epoxy pre-coated wood substrates (samples S3) using a spray gun operating at 0.2 MPa compressed air at a distance of about 10 cm. For comparison purpose, the hydrophobically modified ZnO suspension was also applied to blank wood samples (samples S2). Finally, the coating was dried at room temperature for 8 h.

### 2.4. UV Irradiation Test

The color stability of wood surfaces was determined using a Guanhya UVA 340 lamp with power of 40 W, Shenzhen, Guangdong, China (Mainland), for 900 h in a Laboratory-made UV chamber, at ambient temperature and humidity inside. Color measurement was carried out using an NF-333 spectrophotometer (Nippon Denshoku, Tokyo, Japan) before and after UV irradiation.

The total color change, ∆*E*, was calculated according to the following equations [28],

∆*E* = (∆L*^2^ + ∆a*^2^ + ∆b*^2^)^1/2^

where ∆L*, ∆a*, and ∆b* were calculated according to the following equations,

∆L* = L*_1_ − L*_o_

∆a* = a*_1_ − a*_o_

∆b* = b*_1_ − b*_o_

where L*_o_, a*_o_, b*_o_ and L*_1_, a*_1_, b*_1_ are average values of color coordinates obtained by spectrophotometer on each sample before and after UV irradiation.

### 2.5. Mechanical Resistance Test

Mechanical resistance of the superhydrophobic coating was tested by using a water jet with a distance of 10 cm from the nozzle to the sample surface [29,30]. The samples were tilted at 45 degrees during ejection. The samples were unceasingly exposed to a water jet, having a water pressure of 25 kPa for five minutes. After that, the samples were dried again at room temperature for two hours. The experiments were conducted five times, and the water contact angles (WCAs) were measured after each water jetting.

### 2.6. Characterization Methods

The WCAs on the uncoated and coated wood samples surfaces were measured to test for hydrophobicity. In this experiment, the WCAs were measured on pictures of water droplets (about 5 μL) on the wood sample surface, taken with a digital camera that connected to a computer by ImageJ software (National Institutes of Health, Bethesda, USA) with the Low bond axisymmetric drop shape analysis (LB-ADSA) plugin. The WCAs of five different zones on the wood sample surfaces were measured.

The microstructure morphology of wood sample surfaces was characterized by a field-emission scanning electron microscope (FE-SEM, S-4800, Tokyo, Japan) combined with energy-dispersive X-ray (EDX, Oxford Instruments) spectroscopy at the voltage of 10 kV. The crystal structure of wood samples was analyzed by X-ray diffraction with a SIEMENS X-ray diffractometer, Model D5005.

## 3. Results and Discussion

### 3.1. Microstructure Morphology, Chemical Composition and Crystal Structures of the Coating

Researches in the field of superhydrophobic coating indicated that, in order to obtain superhydrophobic surfaces for materials, a homogeneous and continuous coating is essential. Furthermore, the coating must have micro/nano roughness and low surface energy [31]. Many methods were applied to fabricate superhydrophobic coating based on this theory. In addition, there was much published research with remarkable results in the field of superhydrophobic coating for wood [11,12,32]. However, the durability of the superhydrophobic coating for wood is still the problem that needs to improve. Recently, epoxy resin was used as a bonding material to create a bridge-linking between nanoparticles and substrates in some studies. Results showed that epoxy is a handy binder for improving the durability of superhydrophobic coating [30,33,34].

**Figure 1:**
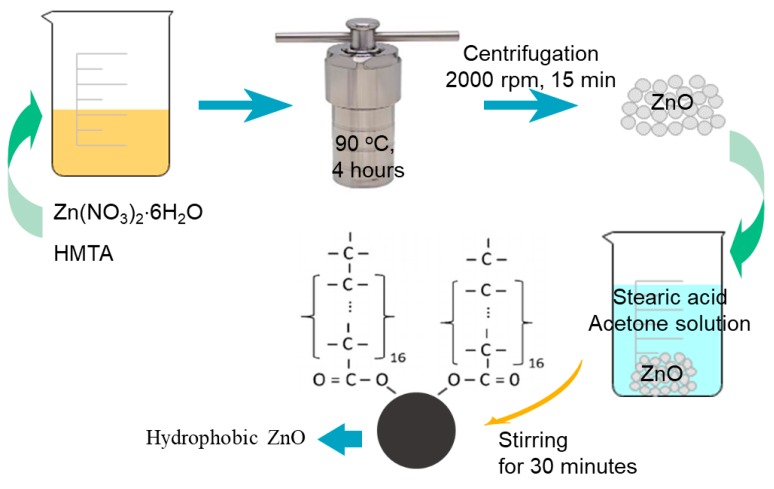
A schematic illustration of the hydrophobic ZnO micro/nanoparticles.

**Figure 2:**
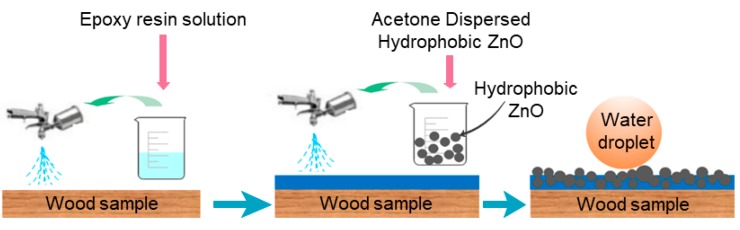
A schematic illustration of the superhydrophobic coating preparation on wood surfaces.

In the current experiment, hydrophobic ZnO particles played a role to make surface roughness as well as to reduce the surface energy. The epoxy was a binding agent that created a bonding between ZnO particles and wood surfaces. Hence, durable superhydrophobic coating for wood has been obtained. The preparation process includes two main steps shown as the diagram in Figure 1; Figure 2. The first step, ZnO particles with micro/nano size were fabricated by a hydrothermal method [27]; however, the hydrophobicity of as-prepared ZnO particles was not high. Therefore, it is necessary to modify ZnO particles, producing highly hydrophobic ZnO particles, even superhydrophobic ZnO particles. Thus, the modification by acetone solution of stearic acid was used to achieve the hydrophobic ZnO particles [25] (Figure 1).

In the second step, the hydrophobic ZnO particles were applied to wood samples by a simple spray coating method using a spray gun (Figure 2). In this step, firstly the samples were pre-coated with epoxy by impregnation to obtain continuous incompletely cured epoxy coating. This incompletely cured epoxy coating will create a binding on wood surfaces. After that, the dispersed hydrophobic ZnO in acetone was then sprayed onto the epoxy pre-coated wood samples. Finally, the coating was dried at room temperature for 8 h to obtain the durable epoxy@ZnO superhydrophobic coating on wood (samples S3). For comparison, a wood sample coated with ZnO hydrophobic particles without epoxy pre-coated was also prepared (samples S2).

#### 3.1.1. Microstructure Morphology

**Figure 3:**
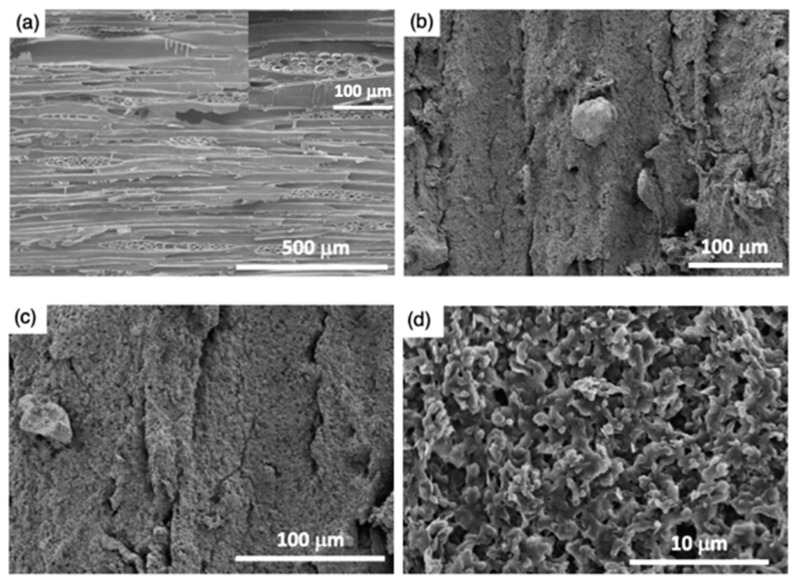
Field emission scanning electron microscopy (FESEM) images of the surfaces of (**a**) uncoated wood (sample S1); (**b**) ZnO coated wood (sample S2); (**c**) epoxy@ZnO coated wood (sample S3); and (**d**) large magnification of sample S3.

The microstructure morphology of uncoated wood (sample S1), ZnO coated wood (sample S2), and epoxy@ZnO coated wood (sample S3) surfaces was characterized by field emission scanning electron microscopy (FESEM). The FESEM image of sample S1 is shown in Figure 3a. It can be seen that the wood surface was constructed by wood tissues such as wood vessels and parenchyma in wood rays. These wood tissues created the wood surface itself large-scale roughness features contain microchannels with 20–200 μm in width. The FESEM image of sample S2 is shown in Figure 3b and the FESEM images of sample S3 with different magnifications are shown in Figure 3c,d. It could be observed that the wood surfaces were coated continuously with a layer of microporous structures of ZnO particles, which formed from the rapid evaporation of acetone (Figure 3b,c). As shown in Figure 3d, upon increasing magnification of FESEM image, the coating possessed a rough surface with hierarchical micro/nanoscale structures. These hierarchical micro/nanoscale rough structures played a significant role in forming the superhydrophobicity.

#### 3.1.2. Chemical Composition

**Figure 4:**
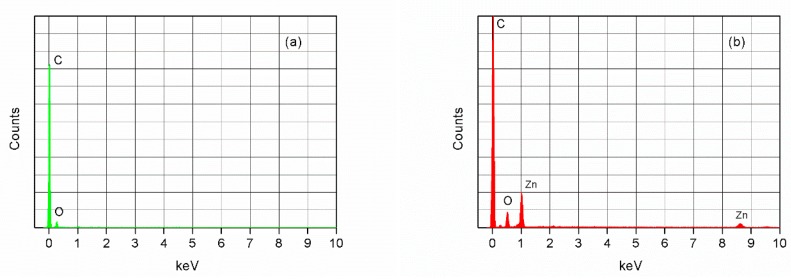
EDX spectra of the (**a**) uncoated wood (sample S1) and (**b**) epoxy@ZnO coated wood (sample S3).

The presence of ZnO in the epoxy@ZnO coating of coated wood samples (S3), as observed by FESEM, was also analyzed by energy-dispersive X-ray analysis (EDX). Figure 4a,b shows the EDX spectra of the uncoated wood surface and epoxy@ZnO coated wood surface, respectively. It can be seen from the spectra that there was a significant zinc (Zn) peak in the spectra of sample S3 (Figure 4b) compared to those of sample S1 (Figure 4a). These results suggest that ZnO was a constituent element present in the coating.

#### 3.1.3. Crystal Structures

**Figure 5:**
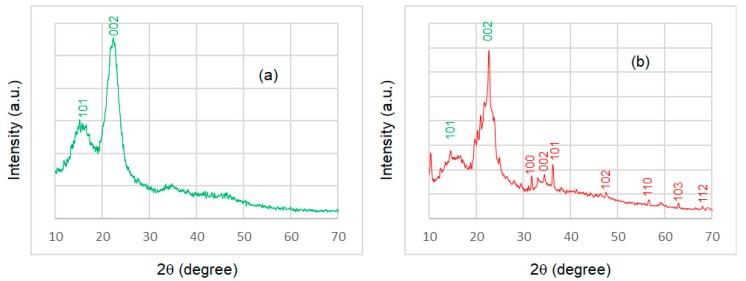
EDX patterns of the (**a**) uncoated wood (sample S1) and (**b**) epoxy@ZnO coated wood (sample S3).

The results of FESEM and EDX analysis proved the zinc element present in the coating. In order to clarify the ZnO crystal structure, an X-Ray Diffraction (XRD) analysis was conducted. The XRD patterns of the uncoated wood sample (S1) and epoxy@ZnO coated wood sample (S3) were collected across a 2θ ranges of 10° to 70° to characterize their crystal structures. The XRD patterns of the uncoated wood sample and epoxy@ZnO coated wood sample are shown in Figure 5.

In the XRD pattern of the sample S1 (Figure 5a), the only diffraction peaks, located at 2θ of 16° and 22.6°, were assigned to peaks of the cellulose crystal planes 101 and 002, respectively [35]. However, in the XRD pattern of sample S3 (Figure 5b), additional diffraction peaks were observed except these peaks of cellulose crystal. These diffraction peaks were assigned to peaks related to the wurtzite hexagonal structure of ZnO (card number 36–1451) [27]. No additional peaks were found in the epoxy@ZnO coated wood sample.

### 3.2. Hydrophobicity of the Coating

The most recognizable definitions in surface science indicate that a surface is regarded as hydrophobic if the water contact angle (WCA) on its surface is larger than 90°; otherwise, it is classified as hydrophilic. Moreover, it can be considered superhydrophobic if the water contact angle on its surface is larger than 150° [36].

**Figure 6:**
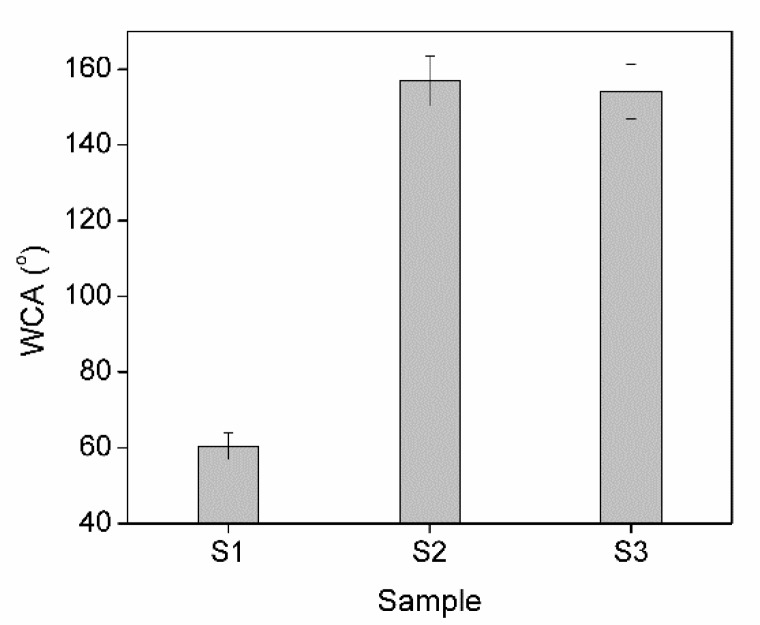
The water contact angle of uncoated and coated wood surfaces. Uncoated wood (S1), ZnO coated wood (S2) and epoxy@ZnO coated wood (S3).

In the present experiment, the hydrophobicity of uncoated wood and the epoxy@ZnO coated wood was characterized by the WCA. As shown in Figure 6, the uncoated wood (samples S1) exhibited a hydrophilic character, with a WCA of 60.4°. After the application of the ZnO coating (samples S2) and epoxy@ZnO coating (samples S3) the wood surfaces became superhydrophobic, with an average WCA of 156.8° and 154.1°, respectively.

The method used to prepare hydrophobic ZnO in this study was almost similar to the method of Xiang et al. [25] with some modifications. The ZnO superhydrophobic micro/nanocoating was prepared by using laboratory self-fabricated ZnO powder followed by stearic acid modification, and then the as-modified ZnO powders were applied on wood surfaces by simple spray coating. Therefore, the obtained superhydrophobic ZnO coating and epoxy@ZnO coating can also be explained.

However, the average WCA of samples S2 was slightly larger than that of samples S3. This phenomenon may be caused by the epoxy pre-coating being applied on surfaces of samples S3. It can be observed from FESEM analysis that there were only layers of hydrophobic ZnO with hierarchical micro/nanoscale structures in the ZnO coating without epoxy pre-coated (Figure 3b). Nevertheless, some particles of hydrophobic ZnO particles in these layers were enveloped by a part of epoxy. So that the hydrophobicity of these ZnO particles was reduced, resulting in the WCA on samples S3 smaller than that of samples S2.

**Figure 7:**
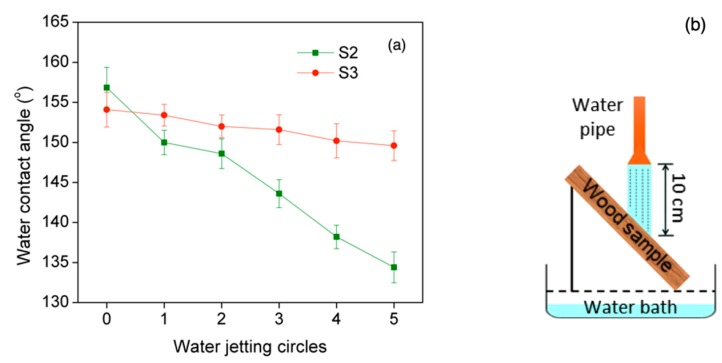
(**a**) The results of water contact angle (WCA) measurement and (**b**) schematic illustration of water jetting test on coated wood surfaces. ZnO coated wood (S2) and epoxy@ZnO coated wood (S3).

Fundamental to establishing superhydrophobicity of coating are hierarchical micro/nanoscale structures. However, these structures are not usually mechanically durable, which restricts the extensive use of superhydrophobic coatings. Recently, in order to improve the robustness of the coating, an adhesive to bond the coating to the substrate was used. In this research, the incompletely cured epoxy pre-coated layer played the role of bonding the hydrophobic ZnO particles to wood surfaces to improve the durability of the mechanically weak ZnO coating. In order to investigate the effect of the epoxy pre-coated layer to the durability of the coating, the water jetting test was carried out on the obtained coating of samples S2 and S3. A schematic illustration of the water jetting test on coated samples and WCA measurement results were shown in Figure 7.

As could be seen in Figure 7, the WCA of samples S2 and samples S3 was reduced by increasing the water jetting circles. However, the reduction of WCA of samples S2 was stronger than that of samples S3. The WCAs of samples S2 varied between 134.4° and 156.8°, the WCAs of samples S3 varied between 149.6° and 154.1° in the five water jetting instances. Experiment results also suggested that the superhydrophobic properties of the samples S2 were destroyed by the water jet easier than that of samples S3, indicating that the added epoxy pre-coating played a significant role in improving the robustness of ZnO superhydrophobic coating. These results are similar to the results of researches that used other types of nanomaterials [29,30,34].

### 3.3. Color Stability during UV Irradiation

**Figure 8:**
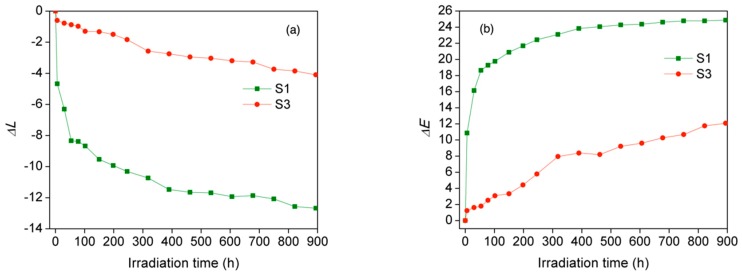
Color changes of (**a**) uncoated wood surfaces and (**b**) epoxy@ZnO coated wood surfaces versus irradiation time; uncoated wood (S1) and epoxy@ZnO coated wood (S3).

The durability of epoxy@ZnO coating on wood surfaces can contribute to sound surface stability, which is necessary for outdoor applications, where the wood surface may be degraded by natural weathering conditions, such as rain, wind and UV-light irradiation. The color stability efficiency of the epoxy@ZnO coated wood surface was evaluated by color measurement after UV exposure. The effect of UV light exposure on uncoated wood surfaces and epoxy@ZnO coated wood surfaces is shown in Figure 8.

As can be observed from the results of *∆L* in Figure 8a, the lightness (L*) of uncoated samples and coated samples was reduced by increasing the irradiation time. The lightness of uncoated samples was reduced rapidly after 84 h of irradiation, and then it was reduced slowly and almost unchanged after about 852 h of irradiation. In contrast, the lightness of epoxy@ZnO coated samples was reduced gradually with increased irradiation time. Moreover, the lightness change of epoxy@ZnO coated samples was smaller than that of uncoated samples. The total color change (*∆E*) is shown in Figure 8b. The *∆E* value of both uncoated samples and epoxy@ZnO coated samples increased with an increase in irradiation time. The obtained results also showed a similar trend of lightness change. The *∆E* value of uncoated samples increased rapidly in around 80 h at of the initial irradiation duration, and reached a mostly constant value after about 800 h of irradiation. The ∆*E* value of epoxy@ZnO coated samples increased slowly and at a lower rate than that of uncoated wood, suggesting that the epoxy@ZnO protected the wood surface against color change caused by UV irradiation.

It is well known that the color changes on the wood surface samples after UV irradiation mainly results from the photo-oxidation of lignin [2]. Nano zinc oxide (ZnO) is known to absorb UV radiation. ZnO nanoparticles can offer UV protection to coatings and underlying substrates. This decreased the intensity of UV light when in contact with wood components, delaying the oxidation of wood surfaces [23,37]. Therefore, the color stability of epoxy@ZnO coated wood was improved compared to that of uncoated wood. The observed phenomenon may be caused by the high UV radiation absorption capability of the wurtzite hexagonal structure of ZnO.

## 4. Conclusions

A epoxy@ZnO superhydrophobic coating was successfully applied on *Styrax tonkinensis* wood by a two-step spray coating method. Both ZnO coating and epoxy@ZnO coating played a part in a continuous epoxy@ZnO coating with hierarchical micro/nanoscale structures constructed by wurtzite hexagonal structure of ZnO micro/nanoparticles. The hydrophobicity of wood was improved after being coated by ZnO coating and epoxy@ZnO coating. The average water contact angle of ZnO coating was a little larger than that of epoxy@ZnO coating. However, the robustness of epoxy@ZnO coating was significantly better than that of ZnO coating after exposure to the water jet, which can be attributed to the high bonding strength of epoxy pre-coating. The color stability of wood coated by epoxy@ZnO coating was significantly improved. The total color changes of epoxy@ZnO coated wood samples were over around 50% greater than that of uncoated wood samples. The enhancement of hydrophobicity and color stability of wood after being coated by epoxy@ZnO coating can be associated with high UV absorption capability of the wurtzite hexagonal structure of ZnO that are present in the epoxy@ZnO coating. The two-step spray coating method of the present research was easy and convenient. Therefore, it can be used for large-scale production that improves hydrophobicity and color stability of wood.
